# Acute Hepatic Allograft Rejection in Pediatric Recipients: Independent Factors

**Published:** 2017-11-01

**Authors:** S. M. Dehghani, I. Shahramian, M. Afshari, M. Bahmanyar, M. Ataollahi, A. Sargazi

**Affiliations:** 1 Division of Gastroenterology, Department of Pediatrics, Shiraz University of Medical Sciences, Shiraz, Iran; 2 Division of Gastroenterology, Department of Pediatrics, Zabol University of Medical Sciences, Zabol, Iran; 3 Department of Community Medicine, Zabol University of Medical Sciences, Zabol, Iran; 4 Student Research Committee, Zabol University of Medical Sciences, Zabol, Iran

**Keywords:** Graft rejection, Immunity, cellular, Liver transplantation, Pediatric

## Abstract

**Background:**

Acute cellular rejection (ACR) has a reversible effect on graft and its survival.

**Objective:**

To evaluate the relation between ACR and clinical factors in recipients of liver transplant allografts.

**Methods:**

47 consecutive liver recipients were retrospectively studied. Their data were extracted from records and analyzed.

**Results:**

38 (81%) of the 47 recipients experienced ACR during a 24-month follow-up. The rate of rejection was associated with none of the studied factors—recipient’s blood group, sex, age, familial history of disease, drugs and blood products received, type of donor, and Child score and class.

**Conclusion:**

During a limited follow-up period, we did not find any association between ACR and suspected risk factors.

## INTRODUCTION

Nowadays, liver transplantation is an accepted treatment for end-stage liver diseases in pediatric patients [1]. The first attempt for liver transplantation was done by Starzel in 1963 on a 3-year-old boy with biliary atresia [2]. Despite of the technological, medical and surgical improvements, liver transplantation is associated with mortality and morbidity. Acute cellular rejection (ACR) is one of the main post-transplantation complications affecting the graft function [3-5].

The incidence of ACR in children following liver transplantation was reported differently from <20% to 50% based on age of recipient (in older children it is more frequent); it can occur at any time after the transplantation [6]. Suspicion for ACR is made by chemical and biochemical evidence and the liver biopsy is mandatory for confirmation of the diagnosis. Histological criteria for ACR are based on Sensor’s triad of portal hepatitis, endotheliitis, and lymphocytic cholangitis [7].

Because of reversibility and the impact of ACR on long-term graft function as well as graft loss, this study was conducted to evaluate the impact of pre-transplantation, concomitant and post-transplantation factors of ACR in recipients of allograft liver.

## MATERIALS AND METHODS

Between 2012 and 2015, 318 pediatric patients with congenital hepatic disease received liver transplantation in Nemazi Hospital affiliated to Shiraz University of Medical Sciences. Complete records of 47 consecutive pediatric patients with ACR were collected and followed for data on sex, age, weight, age at ACR, age at liver transplantation, Child score, blood group, family history of transplantation, operation data, and its complication; also post-transplantation treatment and their circumstances were collected. In this study, the including criteria were having age between 1 and 18 years, having a biopsy-proven ACR, and presence of no other complications. The exclusion criteria were presence of other explanations for the signs, no history of previous liver transplantation, and secondary liver malignancy. Histological characteristics for ACR were lymphocytic portal inflammation, bile duct inflammation, and endotheliitis.

Statistical Analysis

The crude and adjusted associations between potential risk factors and acute rejection were estimated using univariate and multivariate (controlling for treatment delay, Child class, and administration of spironolactone and whole blood transfusion before transplantation) poisson regression models. All statistical analyses were performed with Stata SE ver 11. A p value <0.05 was considered statistically significant.

## RESULTS

A total of 47 patients who underwent liver transplantation was followed for a maen±SD of 1.8±1.9 years. The mean±SD age of participants was 9.6±9.6 years; 25 (53%) patients were female; 33 (72%) received transplant from dead donors, the remaining received transplants from their live parents.

Characteristics of patients with ACR are shown in Tables 1, and 2. Univariate analysis revealed that blood transfusion before transplantation increased the risk of ACR (RR=2.79, p=0.04). However, after adjusting for confounders, in a multivariate analysis, the risk was found to be non-significant (p=0.2) (Table 3).

**Table 1:** Characteristics of patients with acute liver

Factors	n (%)
Sex	
Male	22 (46)
Female	25 (52)
Blood group	
A	17 (35)
B	10 (21)
O	19 (40)
AB	1 (2)
Family history	
No	35 (75)
Yes	12 (26)
Spironolactone before transplantation	
No	25 (53)
Yes	22 (47)
Furosemide before transplantation	
No	40 (85)
Yes	7 (15)
Donor type	
Dead	34 (72)
Father	3 (6)
Mother	10 (21)
Irradiated packed red cells before transplantation	
No	34 (72)
Yes	13 (28)
Whole blood before transplantation	
No	32 (68)
Yes	15 (32)
FFP before transplantation	
<4 units	44 (94)
>4 units	3 (6)

**Table 2:** Mean±SD of some characteristics of patients with acute liver transplant rejection

Factors	Mean±SD
Age (yrs)	8.3±4.4
Weight (kg)	23.7±13.4
Child score	7.7±2.3
Child class	2.0±0.7
Cold ischemia time (min)	14.7±18.3
Warm ischemia time (min)	44.8±13.0
Irradiated packed red cell	0.6±1.5
FFP	1.1±1.8
Follow-up period (yrs)	2.2±1.9
Treatment delay	4.0±3.4

**Table 3:** Crude and adjusted relative risks of acute rejection for different factors before and after transplantation

Factors	Crude RR (95% CI)	Adj RR (95% CI)
Irradiated packed red cells before transplant	1.28 (0.58–2.82)	3.84 (0.89–16.62)
Irradiated packed red cells during surgery	1.00 (1.00–1.00)	1.00 (0.99–1.01)
Whole blood before transplant	2.79 (1.04–7.52)	3.19 (0.48–21.07)
FFP before surgery	0.72 (0.22–2.34)	1.72 (0.40–7.36)
FFP during surgery	1.03 (0.96–1.10)	1.29 (0.77–2.17)
Blood group (compared with A)		
B	0.72 (0.31–1.68)	0.36 (0.08–1.59)
O	0.61 (0.29–1.29)	0.38 (0.10–1.36)
AB	0.59 (0.08–4.52)	0.60 (0.06–5.92)
Sex	1.40 (0.74–2.64)	1.33 (0.39–4.53)
Age	1.01 (0.95–1.06)	0.93 (0.70–1.24)
Weight	0.99 (0.96–1.02)	1.00 (0.93–1.08)
Familial history	0.86 (0.35–2.12)	1.01 (0.21–4.85)
MELD-PELD	0.98 (0.94–1.03)	1.01 (0.90–1.15)
Child score	0.95 (0.81–1.11)	0.66 (0.28–1.52)
Child class	0.57 (0.29–1.10)	0.78 (0.22–2.73)
Furosemide before surgery	0.92 (0.38–2.20)	1.97 (0.30–13.01)
Spironolactone before surgery	0.56 (0.29–1.07)	0.85 (0.20–3.54)
Albumin before surgery	0.90 (0.71–1.14)	1.24 (0.68–2.27)
Donor type (compared with dead)		
Father	1.11 (0.34–3.68)	1.25 (0.13–11.50)
Mother	0.62 (0.28–1.36)	0.50 (0.09–2.61)
Cold ischemia	1.00 (0.98–1.01)	0.99 (0.96–1.02)
Warm ischemia	1.00 (0.97–1.02)	0.99 (0.94–1.04)
Infection	1.02 (0.14–7.46)	1.28 (0.17–9.68)
Drug allergy	2.96 (0.40–21.58)	2.96 (0.40–21.58)
Treatment delay	1.03 (0.99–1.07)	1.01 (0.83–1.22)

## DISCUSSION

In this study, we observed 47 cases of ACR among 318 liver transplant recipients. We found that ACR was not associated with any of the factors investigated before and after the surgery. Multivariate analysis, showed that only receiving irradiated packed red cells before surgery had a borderline significant association with ACR—receiving irradiated packed red cells caused approximately a four-fold increase in the risk of rejection. Wang, *et al*, conducted a retrospective study for evaluation of ACR risk factors on 110 consecutive liver recipients and found a significant relation between recipients age and ACR. Their explanation for this association was a higher CD8 lymphocyte count in younger patients [8]. Shindoh, *et al*, performed a prospective study on records of 413 patients who received graft from living donors. They concluded that autoimmune liver diseases are associated with higher risk for ACR and its relapse [9].

Fan, *et al*, compared the incidence and severity of ACR between living-donor and cadaveric liver transplant recipients. They found no significant difference between groups; also, HLA mismatch and cold ischemic time had no impact on the incidence and severity of ACR [10]. These finding are in keeping with those observed in our study. Toyoki, *et al*, evaluated the immunological advantage of living-related liver transplantation compared with cadaveric transplants in 100 pediatric liver recipients. They confirmed a comparable incidence of ACR between groups in the first 24 months of transplantation. However, ACR episodes after one year were significantly lower in living-related liver recipients [11]. Similar to our results, in a systemic review and meta-analysis carried out by Hu, *et al*, ACR and graft survival rates were not related to donor types (alive or cadaveric) [12]. Sandra, *et al*, in their study on 114 pediatric patients reported a higher incidence of ACR in maternal grafts with sex mismatch [13], which was in contrast to the results of the current study.

Kinpan and his colleagues retrospectively investigated the information of 778 liver recipients to assess the impact of clinical factors on ACR incidence. They concluded that old age, chronic hepatitis B, living donor, and use of iterleukin-2 receptor agonists were associated with lower incidence of ACR [14]. Unfortunately, we could not record the exact date of many events. Therefore, survival analysis was not possible for our data; only the associations were investigated during a limited follow-up period from the each participant entry to March 2016. Moreover, because our data were collected and analyzed retrospectively, some necessary data might be ignored during the study. 

In conclusion, our study did not find any risk factors for liver transplant rejection. Further multicenter cohorts with larger sample size and longer follow-up period are required to identify the independent risk factors of transplant rejection after liver transplantation. 
